# Bluetooth Device Identification Using RF Fingerprinting and Jensen-Shannon Divergence

**DOI:** 10.3390/s24051482

**Published:** 2024-02-24

**Authors:** Rene Francisco Santana-Cruz, Martin Moreno-Guzman, César Enrique Rojas-López, Ricardo Vázquez-Morán, Rubén Vázquez-Medina

**Affiliations:** 1 Instituto Politécnico Nacional, Centro de Investigación en Ciencia Aplicada y Tecnología Avanzada, Santiago de Querétaro 76090, Mexico; rsantanac2200@alumno.ipn.mx; 2 Universidad Tecnológica de San Juan del Río, San Juan del Río 76800, Mexico; mmorenog@utsjr.edu.mx; 3 Instituto Politécnico Nacional, Escuela Superior de Ingeniería Mecánica y Eléctrica, San Francisco Culhuacan, Mexico City 04440, Mexico; crojas@ipn.mx (C.E.R.-L.); rivazquezm@ipn.mx (R.V.-M.)

**Keywords:** identification systems, radio frequency fingerprints (RFF), IoT device identification, cybersecurity, wireless communication

## Abstract

The proliferation of radio frequency (RF) devices in contemporary society, especially in the fields of smart homes, Internet of Things (IoT) gadgets, and smartphones, underscores the urgent need for robust identification methods to strengthen cybersecurity. This paper delves into the realms of RF fingerprint (RFF) based on applying the Jensen-Shannon divergence (JSD) to the statistical distribution of noise in RF signals to identify Bluetooth devices. Thus, through a detailed case study, Bluetooth RF noise taken at 5 Gsps from different devices is explored. A noise model is considered to extract a unique, universal, permanent, permanent, collectable, and robust statistical RFF that identifies each Bluetooth device. Then, the different JSD noise signals provided by Bluetooth devices are contrasted with the statistical RFF of all devices and a membership resolution is declared. The study shows that this way of identifying Bluetooth devices based on RFF allows one to discern between devices of the same make and model, achieving 99.5% identification effectiveness. By leveraging statistical RFFs extracted from noise in RF signals emitted by devices, this research not only contributes to the advancement of the field of implicit device authentication systems based on wireless communication but also provides valuable insights into the practical implementation of RF identification techniques, which could be useful in forensic processes.

## 1. Introduction

In the realm of IoT ecosystems, especially those characterized by high device density, Bluetooth is emerging as the first choice for wireless communication among IoT gadgets. This preference is due to Bluetooth’s minimal energy requirements, the cost-effectiveness of its radio components, and its innate ability to establish direct connections with smartphones, thus providing a seamless interface for users [1,2,3]. Concurrently, the development and integration of sophisticated RFF methods for IoT devices are underscored by the critical need for identifying and mitigating unauthorized access. This capability not only strengthens the security framework of network infrastructures, but also protects against intrusion into critical systems [4,5]. In alignment with these security measures, RFF is recognized for its pivotal role in strengthening the security of wireless devices [6]. By leveraging unique identifiers, RFF enhances the accuracy of device recognition, significantly reducing the risks associated with spoofing attacks and thereby strengthening the resilience of network defenses. IoT applications use the 2.4 GHz ISM band of Bluetooth technology, referred to as RF signals, for short-range communication [7,8,9]. The technology has difficulty distinguishing individual devices within the radio frequency spectrum, requiring the use of advanced identification techniques. These techniques typically involve machine learning approaches.

The adoption of machine learning for RF device identification, including SVM and deep learning, faces significant challenges. These methods require extensive and varied datasets, which are often scarce. The Multisampling Convolutional Neural Network (MSCNN) for RFF has demonstrated high accuracy in IoT device identification, achieving up to 97.00% under line-of-sight conditions [10]. However, it requires large datasets and may encounter scalability issues due to model complexity. CNNs for RFF offer scalability and rich feature-extraction capabilities, but exhibit inconsistent performance in different environments, with accuracy dropping to below 78.00%, and demanding high computational resources, making them unsuitable for real-time applications in resource-constrained environments [11]. The Full Neural Network (FNN) distinguishes NFC tags with up to 96.16% accuracy, outperforming both CNN and RNN in NFC security applications [12]. Conversely, the Dense Neural Network (DNN) achieves 98.69% accuracy in RFF, although its effectiveness is highly dependent on the quality and size of the images, indicating potential limitations under varying conditions [13]. This highlights a trade-off between accuracy and resource requirements, underlining the need for optimization to improve applicability. Additionally, the lightweight CNN for Zigbee device identification records an accuracy of 93.47% in low SNR conditions, questioning its robustness [14]. CNNs that incorporate multi-channel inputs and feature fusion represent an improved approach, offering better identification accuracy and robustness [15].

SVMs and Neural Networks (NN) have been scrutinized, with SVM using a polynomial kernel function to achieve higher accuracy, in particular demonstrating remarkable effectiveness in Bluetooth device identification with a classification accuracy of 97.90% [16,17,18]. Nevertheless, the reliability of SVM in Bluetooth RFF is questioned at low Signal-to-Noise Ratio (SNR) levels, showing a reduced accuracy of 90.53% and highlighting concerns in weak signal scenarios for network security applications [19]. The use of SVMs, while advantageous for dealing with high-dimensional data and nonlinear relationships through the kernel, encounters skepticism due to the complexity of kernel selection, parameter tuning, scalability issues, and limited interpretability, challenging their feasibility in large-scale RFF systems [20]. Moreover, an accuracy of 94.20% in some RFF cases indicates potential overfitting, computational intensity, and the complexity of hyperparameter tuning, which could limit their practicality [21]. Additionally, while achieving a 90.00% accuracy in RFF for device-to-device (D2D) security, concerns remain regarding scalability and kernel selection, which could impact performance in various scenarios [22]. A study using Variational Mode Decomposition (VMD) for feature extraction with Linear SVM (LSVM) showed an improved accuracy of 97.10% for Bluetooth device classification at lower SNR levels, suggesting that extracting Higher Order Statistics (HOS) features could enhance classification performance by approximately 4%. However, the effectiveness of this method decreases significantly below certain SNR thresholds, highlighting the limitations in noisy conditions [23]. The innovative RF-DNA and SVM-based approach for IoT security, despite its high complexity, faces critical implementation challenges for effective robust device authentication due to its 93.20% accuracy rate, making it a resource-intensive solution [24].

This paper introduces a novel RFF technique derived from intrinsic noise and analyzed using the Probability Density Function (PDF) specifically for Bluetooth devices in IoT applications. The innovation of this study lies in its simplicity and the unique application of the PDF of RFF for Bluetooth device classification, using the JSD for enhanced accuracy. Unlike previous studies that have used RFF, none have applied the PDF of RFF to Bluetooth devices, which marks a distinctive approach in this research. Central to this method is the collection of noise reference signals during the steady-state operation of Bluetooth devices, collected at the receiver end in a controlled experimental setup. This setup is essential to minimize external interference and ensure data the consistency and reliability, and to accurately represent the unique RF emissions of each Bluetooth transmitter. The PDF of each noise signal is then calculated, providing a statistical profile of the noise level distribution within the signal, which serves as a unique RFF for each transmitter. The process culminates in the calculation of a concatenated PDF, representing the statistical RFF of the device. By using JSD to compare the probability distributions of disputed signals and RFFs, the method achieves a remarkable device identification accuracy of 99.50%, demonstrating the potential of this approach for accurate Bluetooth device identification in IoT applications.

The rest of this paper is structured into six sections. Section 2 defines the five specifications for device RFFs: uniqueness, universality, persistence, collectability, and robustness. These criteria ensure that each RFF is unique, applicable to a wide range of devices, stable over time, easy to collect and robust under various conditions. Section 3 delves into the specifics of Bluetooth signal processing. It covers the necessary steps, such as signal filtering, state detection, and the RFF definition based on noise signals in Bluetooth communication. In Section 4, a case study is presented, using the noise signal database developed by Uzundurukan et al. [16,17]. This section introduces an MSE-based criterion for determining the number of reference noise signals to be evaluated to set up a Bluetooth RFF. It also proposes a method to compensate for the amplitude difference of noise signals received at different distances between the receiver and the transmitter. The section culminates in demonstrating the practical application of the estimated RFF for device discrimination using JSD. Section 5 provides a critical analysis of the results, comparing the proposed discrimination method with Uzudurukan’s method when applied to the same case study. Finally, Section 6 gives the conclusions of this paper.

## 2. RFF Specifications

The study of device identification based on RFFs defined from noise signals in the Bluetooth frequency band has been a central topic in wireless communication research [10,11,12,13,14,15,16,17,19,20,21,22,23,24,25,26,27,28,29]. Numerous influential studies have significantly advanced on this topic, using various methods and innovative approaches to improve the precision and dependability of RFF techniques [10,11,12,14,15,17,22,25]. A Bluetooth signal can be treated as an investigative object to identify potential threats or attacks. In this context, RFF is a promising technique for secure device discrimination, identification or authentication. According to Soltanieh et al. [6], RFF methods are scrutinized against five fundamental specifications to ensure their efficacy and reliability as follows:

- Uniqueness. It ensures distinctiveness by preventing any two devices from sharing identical RFF, thus facilitating individual device identification.
- Universality. It guarantees unique RFF features for each device, providing complete coverage of all devices on a given network.
- Persistence. It requires the RFF to remain constant over time, unaffected by environmental fluctuations, ensuring stability and reliability in device identification.
- Collectability. It requires that the RFF be quantitatively measurable, allowing for accurate data analysis and device identification using rigorous measurement techniques.
- Robustness. It preserves the integrity of the RFF against environmental changes and device-related factors, ensuring consistent and reliable authentication regardless of varying conditions.

## 3. Bluetooth Signals for the Device Discrimination

This section focuses on the intricate realm of Bluetooth signal manipulation, exploring key techniques such as signal filtering, state identification and RFF. Rooted in the meticulous analysis of noise signals in Bluetooth transmissions, these procedures are fundamental components of understanding and effectively manipulating Bluetooth signals. This section highlights the nuanced methods used to navigate the complexities of Bluetooth signal processing, providing insights essential for further exploration and application in the field.

### 3.1. Noise Model

The analysis of the noise model is defined as:

(1) $$\eta =\eta_{Tx}+\eta_{Rx}.$$

This equation represents the total noise received, η, from a Bluetooth transmitter. The term $\eta_{Tx}$ denotes the intrinsic noise from the transmitter under study, while $\eta_{Rx}$ denotes the additional noise introduced from the receiver. This model is crucial for analyzing and understanding the characteristics of the composite noise in Bluetooth communication. Then, solving Equation (1) yields Equation (2), which allows one to isolate the transmitter-specific noise signal from the total noise.

(2) $$\eta_{Tx}=\eta -\eta_{Rx}.$$

**Figure 1:**
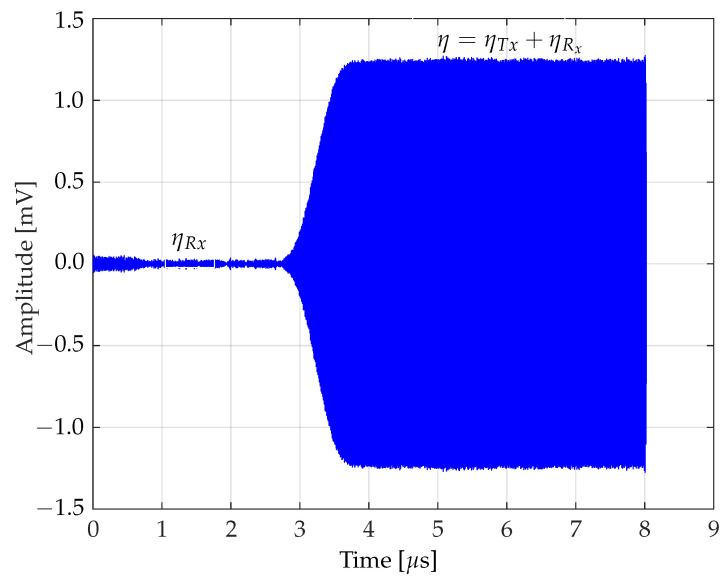
Noise model dynamics in Bluetooth communication systems.

Figure 1 visually presents the noise model as described in Equation (2). It is noteworthy that in the initial state, the noise mainly comes from the receiver *Rx*. However, during the transient state, the transmitter *Tx* is activated, and total noise then embodies both the transmitter and receiver noise components. This transient state gradually stabilizes, reflecting the integration of *Tx* and *Rx*, and thus transitions to a stable state. This visual representation helps to understand the dynamic interaction between transmitter and receiver noise within the Bluetooth communication system.

This analysis describes the interaction between the intrinsic noise of the transmitter $\eta_{Tx}$ and the additive noise from the receiver $\eta_{Rx}$, and their impact on the total noise η. It analyzes how noise dominance shifts from the receiver to the transmitter during a transitional phase, culminating in a combined transmitter-receiver noise state. This approach provides insight into the dynamics of noise in Bluetooth systems.

### 3.2. Signal Filtering

**Figure 2:**
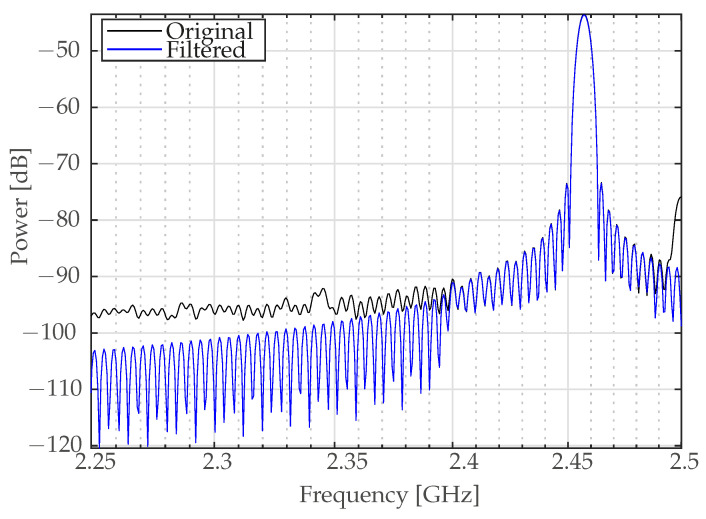
Spectral analysis of bandpass filtered signals in the frequency domain.

Because the signals received at the Bluetooth receiver span contain a variety of components over a wide frequency spectrum, it is required to apply a bandpass filter. This filter is specifically designed to isolate and extract the Bluetooth ISM band from 2.4 GHz to 2.485 GHz (as depicted in Figure 2). In addition, the choice of sampling frequency for this process must adhere to the Nyquist theorem. This theorem states that the sampling frequency must be at least twice the highest frequency component of the signal in order to accurately reconstruct the signal without aliasing. Therefore, the sampling frequency was chosen to be equal to or greater than 4.97 GHz to ensure the integrity and fidelity of the Bluetooth signal. Note that Figure 2 shows two different signals. The first signal shows the wide range of frequencies in the original, unfiltered signal. The second signal shows the ISM band, illustrating how the bandpass filter narrows the frequencies down to only those present inside the Bluetooth band.

### 3.3. State Detection

**Figure 3:**
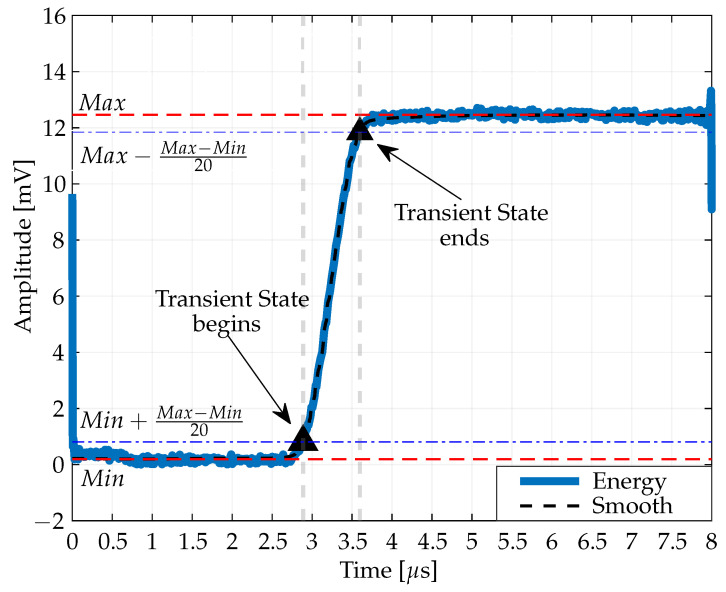
Quantitative assessment of signal state identification and detection from the reference transient state.

Figure 3 provides a clear representation of the different states of a Bluetooth signal as it is received by a Bluetooth receiver. The signal is divided into three distinct parts: the initial state, the transient state, and the steady state. The transient state marks the transition of the signal from its initial state to a steady state. The transient state is identified by specific changes in the signal amplitude. It begins when the signal amplitude is just above its lowest level.

This starting point is mathematically defined as $Min-\frac{Max-Min}{20}$, where Min and Max are the minimum and maximum values, respectively, of the smoothed envelope of the Bluetooth signal. In contrast, the end of the transient state is marked when the signal’s amplitude approaches, but does not quite reach, its maximum level. This end point is described by the expression $Max-\frac{Max-Min}{20}$. This expression helps to determine exactly when the signal goes from changing to reaching a steady state. In summary, Figure 3 is a tool for analyzing a Bluetooth signal, providing a guide to follow the progression of the signal through its initial, transient and steady states, with special emphasis on the transient state because of its significant effect in highlighting the change between the initial and steady states of the Bluetooth signal.

### 3.4. RFFs for Bluetooth Devices

**Figure 4:**
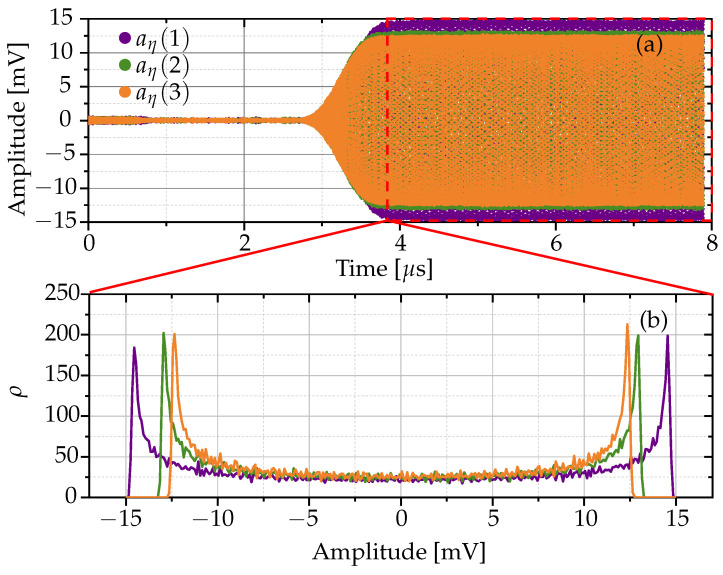
Steady state using three signals on a single device: (**a**) signal analysis in time domain, and (**b**) PDF projection derived from (**a**).

To create the RFF, it is necessary to concatenate a set of noise signals extracted from the steady state of the RF signals at the Bluetooth receiver for each Bluetooth transmitter. This set of noise signals forms the noise reference signals, which are extracted from the Bluetooth signal at the receiver under the assumption that these signals are collected in a controlled experimental setup. For each noise signal in this set, its PDF is calculated. The median PDF, derived from the average of all calculated PDFs, constitutes the statistical RFF to be used in this work. It should be noted that each PDF of the noise signal is calculated using Equation (2), and this process is visually demonstrated in Figure 4.

**Figure 5:**
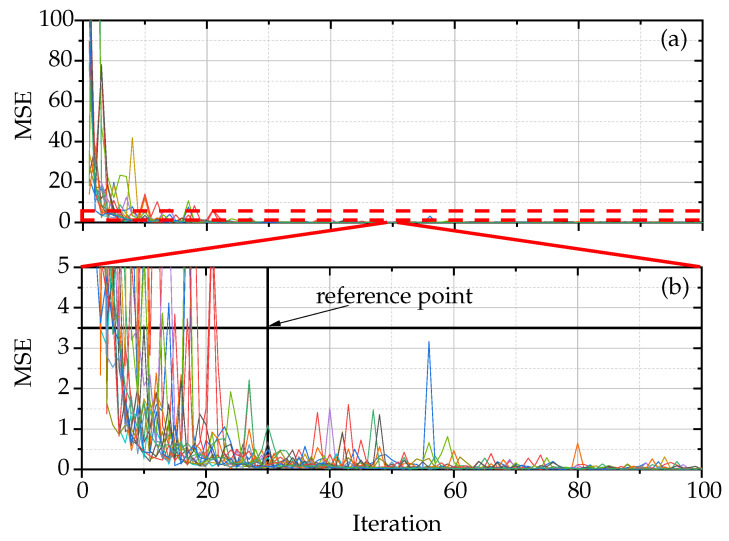
Convergence analysis of the estimated PDF by MSE comparison for *n* and n+1 noise signals. (**a**) Analysis over the full signal range, and (**b**) detailed zoom in on (**a**).

In signal analysis, especially when dealing with noise reference signals in Bluetooth communication, a crucial step is to identify the optimal number of these signals needed for accurate analysis. This process is guided by the MSE criterion, a statistical method for evaluating the accuracy of signal representation. The method involves a comparative analysis of the PDF computed using *n* noise reference signals against the PDF obtained using n+1 noise reference signals. The aim here is to find the point at which increasing the number of signals does not significantly improve the accuracy of the PDF representation. However, the number of noise reference signals that are required should be determined. For this purpose, the MSE criterion was applied between PDF with *n* noise reference signals and PDF with n+1 noise reference signals. According to Figure 5, thirty noise reference signals were considered assuming that MSE is less than 3.5. Note in Figure 5a that the MSE has been calculated from *n* = 2 up to *n* = 100 and when n≥30, the MSE is very small compared to the initial values when n<30. As depicted in Figure 5, a critical threshold is identified at 30 noise reference signals. This decision is based on the assumption that an MSE value of less than 3.5 is acceptable for accurate signal representation. When examining the MSE values for *n* ranging from 2 to 100, as shown in Figure 5a, a notable pattern emerges. It is observed that once the number of noise reference signals reaches 30 or more, the MSE decreases to a level significantly smaller than its values for n<30. This implies that beyond the count of thirty signals, the gain in accuracy, as quantified by the MSE, reaches a plateau, suggesting that additional signals provide minimal improvement.

## 4. Discrimination of Bluetooth Devices

This section delves into the case study discussed and presents a basic scaling method for data analysis and interpretation. It also defines a statistical RFF to uniquely identify each device in the study. Using the JSD, the devices are distinguished by their statistical RFFs, and a detailed description of the methodological framework and the tools used in the analysis is provided.

### 4.1. Case Study

The study uses a comprehensive database of Bluetooth devices, meticulously prepared and compiled in 2020 by Uzundurukan et al. [16]. This database encompasses eight distinct models from four different smartphone brands, as detailed in Table 1. The methodology adopts a systematic approach for device designation, categorizing smartphone name-1 as the original and smartphone name-2 as the corresponding twin. The inclusion of a wide range of models and brands facilitates a comprehensive analysis, and ensure the study’s completeness in examining the distribution and characteristics of Bluetooth devices.

In the database, each smartphone model is paired with a twin variant, leading to a total of sixteen devices. A significant number of signals, specifically one hundred and fifty unique signals per device, are available for analysis. All signals in the database were sampled at a high frequency (5 Gsps), ensuring the acquisition of high-resolution data. This results in a comprehensive collection of two thousand four hundred signals, providing a rich and varied dataset for the study.

### 4.2. Bluetooth Signal Matching

In this work, a statistical method is proposed to define RFF for Bluetooth devices. Statistical characteristics, such as the mean or standard deviation, although commonly used, are often insufficient to provide insight into the behavior of a device. Therefore, the PDF is selected for further analysis. The PDFs calculated for three noise signals in the steady state from a single device are shown in Figure 4. This representation carefully considers the integration of receiver noise with the noise generated by the Bluetooth transmitter, providing a nuanced view of the signal characteristics unique to the device.

In order to minimize potential errors, a scaling method has been proposed, as detailed in Equation (3). It is designed to normalize the amplitude of signals received from different transmitters. In this framework, a(i) is defined as the signal detected at the receiver by monitoring the transmitter *i*; $a_{MAX}\left(i\right)$ represents the maximum amplitude of a(i) and $a_{MAX}\left(1\right)$ indicates the highest amplitude of a(1). In particular, the transmitter signal 1, when monitored, is used as the reference point for scaling. Note, however, it should be noted that any other signal can be used as a reference in this scaling process.

(3) $$a_{e}\left(i\right)=\frac{a(i)}{a_{MAX}\left(i\right)}\cdot a_{MAX}\left(1\right)$$

**Figure 6:**
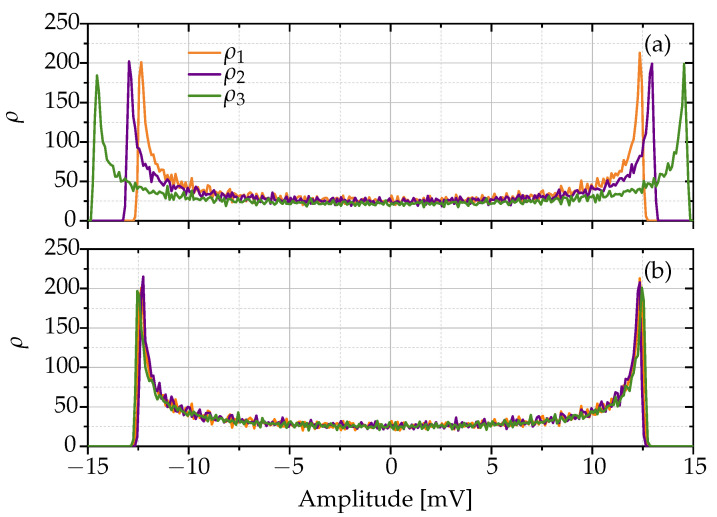
PDF of three noise signals from a single Bluetooth device. (**a**) Before scaling and (**b**) after scaling.

In the graphs of Figure 6, a general overview of the scaling method used is noted. Figure 6a shows a visual representation of the PDF of the three unique noise signals from a single Bluetooth device before the scaling method has been applied. Note the inherent amplitude variation found between PDFs of noise signals of that device. However, Figure 6b demonstrates the result of the applied scaling. Note that the PDFs of the three unique noise signals are more uniform and consistent with each other, showing the ability of the method to standardize the signal characteristics.

### 4.3. Statistical RFF for Case Study

**Figure 7:**
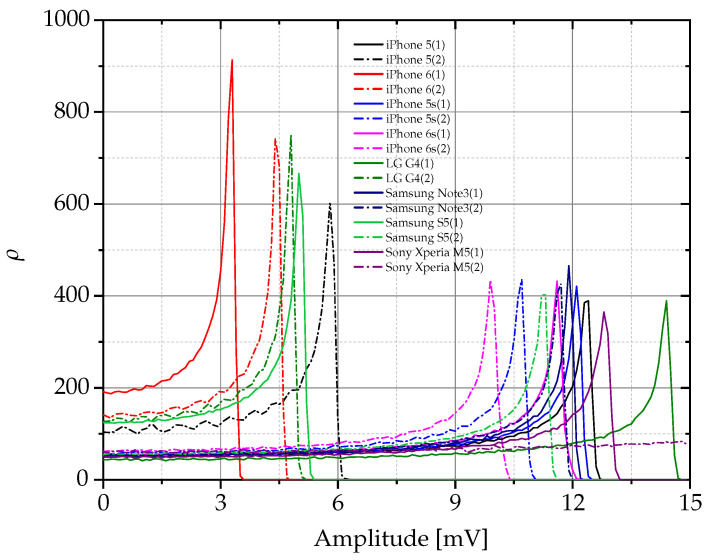
Characterization and analysis of unique RFF patterns from multiple Bluetooth devices.

Figure 7 illustrates the results obtained from the sixteen devices that were examined in the case study. It is important to note that only the positive part of the PDFs of the noise signals was considered. This decision is based on the bimodal and symmetric properties of these statistical distributions. It should be noted that each RFF associated with the devices is unique. This uniqueness of the RFFs accentuates the individual characteristics of each device signal, highlighting the distinctiveness and specificity that the RFF methodology brings to the analysis of device signals. This distinctiveness is useful for understanding and identifying the unique aspects of each noise signal profile within the broader context of the study.

### 4.4. Device Identification by Using Statistical RFF and JSD

For this work, the JSD was used to discriminate the Bluetooth devices in the process of defining a RFF for each, according to Section 3.4. An integral part of this methodology is the use of fifty dispute signals for each device in the discrimination process. The signal that provides the lowest JSD, calculated according to Equation (4), is then assigned to the respective device. The disputed PDF *P* is compared to the reference PDF, which is the RFF extracted from the noise component of the RF signal emitted by a device *Q*. Using the JSD given in Equation (4) for this comparison ensures that disputed signals are assigned to their respective devices, thereby increasing the accuracy of device identification within the study.

(4) $$JSD\left(P\|Q\right)=\frac{1}{2}KLD\left(P\|M\right)+\frac{1}{2}KLD\left(Q\|M\right)\;,$$

where $M=\frac{1}{2}\left(P+Q\right)$.

The findings of this research are presented in Table 2, which shows the confusion matrix derived from the case study. To construct the confusion matrix, the 50 disputed signals are taken into account. Once all the classifications have been made, the model’s accuracy percentage is calculated. This percentage is obtained by dividing the total number of correct predictions by the total number of predictions made and multiplying it by 100 to express it as a percentage. To obtain the effectiveness percentage, the values on the main diagonal of the confusion matrix are averaged. This matrix reveals an accuracy rate of 99.5%. Within this matrix, a notable discrepancy is observed, with an 8% error rate in distinguishing between devices twelve and seven. This specific issue is further explored in Figure 7, which shows the RFF of both devices, in addition to a signal that was assigned to the incorrect device.

The cause of this misattribution can be attributed to the peculiarities of the disputed signal. In particular, its oscillation pattern appears to be the key factor in the error. This pattern differs significantly from the standard patterns observed in the RFFs of these devices. Normally, RFFs are expected to be consistent and distinctive for each device, allowing for accurate identification. However, when a signal exhibits atypical patterns, such as unique oscillations that are not characteristic of the device’s standard RFF, it can lead to misidentification.

## 5. Discussions and Comparisons

In this section, the discussion focuses on the analysis of the results obtained in the previous section of the study. The aim is to delve deeper into the findings and provide a deep understanding of their implications and meaning within the context of the study. The methodological approach and results of Uzundurukan et al. [16] will also be examined in detail in Section 5.2.

### 5.1. Discussion

The results achieved in this study strengthen the concept of developing systems for authenticating individuals using mobile devices identified within an IoT network. In this context, the findings facilitate the creation of algorithms tailored to uniquely identify devices. These algorithms are capable of identifying devices in IoT networks using unique identifiers such as MAC addresses, communication patterns, or unique signatures/RFFs that are both universal and unique to each device. Such technological advances are crucial to strengthening the security of networked environments, where the accurate authentication and identification are essential to prevent unauthorized access and to maintain network integrity, thereby guaranteeing the security and reliability of interconnected systems.

**Figure 8:**
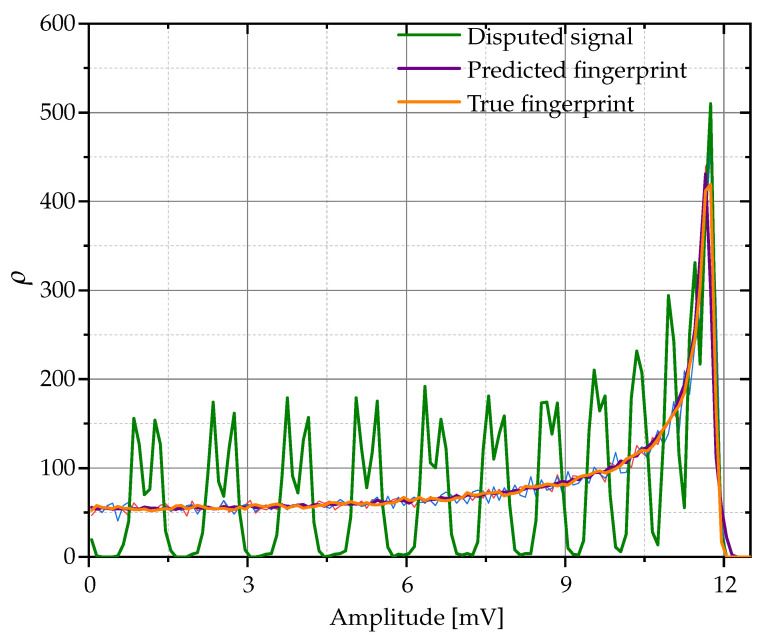
Error analysis in case of signal confusion by comparison of PDFs of RFF signals extracted from Bluetooth devices.

The proposed scaling method for signals in the database is introduced to address the amplitude disparities among the sampled signals. These disparities may arise due to various factors, with the variation in the acquisition distance of each signal being a primary candidate. In an environment where signals are captured from different distances, it is likely that the amplitude of the sampled signal will vary accordingly. It is noteworthy that although some signals were observed to be discretized, they were not discarded in the analysis. The rationale behind this decision lies in the fundamental assumption of the study that all signals are properly acquired. In this context, it is assumed that the differences identified between the signals are due exclusively to variations in the distance between the Bluetooth transmitter and the receiver, rather than errors in the data acquisition process. In Figure 8, an example illustrating the implications of these variations in signal classification is shown. A signal has been incorrectly assigned to a different device due to this discrepancy. This misclassification is suggested to stem from the fact that the signal in question was discretely sampled. This discrete sampling is manifested in the oscillations observed in the dispute signal, implying a non-continuous representation of the data, which may impact the accuracy of the classification process.

### 5.2. Device Identification by Uzundurukan’s Method

A comprehensive comparison is made between the results obtained with the proposed method and with Uzundurukan’s method applied to the case study defined in Section 4.3. This comparison not only evaluates the effectiveness but also takes into account the implementation time, which includes the resources and time required for both the training process and the identification phase. According to Uzundurukan et al. [16,17], Uzundurukan’s method uses a nonlinear SVM with a quadratic kernel, and it applies a process to scale the amplitude of the noise signals similar to that used in the proposed method and detailed in Section 4.2. Although they reported that their SVM-based method achieved an accuracy rate of 97.9%, it could be determined that this accuracy rate was 80.13% when applied to the case study mentioned in Section 4.1. These results, shown in Table 3, allowed us to establish a baseline for the comparative analysis. The discrepancy found could be attributed to the fact that the original method uses the transient state of the noise signals, while the current application focuses on their steady state. First, it is assumed that the noise signal extracted from the transient state is analyzed against the RFF using a SVM. The discrimination process of the RFF consists of two main stages: (a) the training phase, in which the SVM model is applied to a dataset representative of the noise signal using the transient state of the noise signals, allowing the SVM model to learn and adapt, and (b) the discrimination phase, in which the trained RFF is used to analyze new signals and evaluate their congruence with the trained model. This bifurcated methodology, which integrates both training and practical application, facilitates effective device identification.

Uzundurukan’s SVM approach integrates three statistical metrics—kurtosis, skewness, and variance—of the intrinsic noise to provide a comprehensive understanding of system behavior within a pre-trained SVM framework. This SVM was developed using a quadratic kernel when running in MATLAB R23b on an AMD Ryzen 5 2500U computer, with 32 GB of RAM and an AMD Radeon TM Vega 8 processor. This approach provides a global perspective on system statistical dynamics. In contrast, the proposed method uses the PDF of the intrinsic noise to facilitate a detailed examination of system behavior. By using the JSD for comparison against a reference PDF, the method enables a granular assessment. This emphasis on detailed statistical analysis enhances the accuracy of the proposed method. Importantly, both approaches focus their analysis on the steady state of the noise signal, ensuring consistency and reliability in the evaluation process.

## 6. Conclusions

This study concludes that the RFF can be accurately defined by using the PDF of RF noise signals, which are carefully collected from each Bluetooth device in a well-controlled laboratory environment. The statistically derived RFFs satisfy the fundamental characteristics of a device RFF, including aspects such as uniqueness, universality, persistence, collectability, and robustness. In addition, the application of JSD to the PDFs of noise signals, especially in the steady state of the RF signal received by the Bluetooth receiver, and their subsequent comparison with the RFFs of corresponding Bluetooth devices, has proven effective for device classification. The proposed statistical classifier shows promise for broad applicability across different radio frequency technologies, requiring only the computation of statistical distributions of noise signals according to their operating frequency bands. The results of this study clearly highlight the improved efficiency of the proposed statistical method compared to the machine learning approach advocated by Uzundurukan et al. [17]. Remarkably, the statistical classifier achieves a processing time of only 0.21 s, significantly outperforming the machine learning method based on a nonlinear SVM, which requires about 5.35 s. This significant discrepancy in processing time underscores the exceptional speed and computational efficiency of the statistical model, making it particularly advantageous for scenarios requiring rapid data processing. The findings in this work underscore the practical utility of the statistical methodology, especially in situations where time-sensitive data processing is essential for decision making or real-time applications. The efficiency of the statistical classifier, as evidenced by its fast computational capabilities, also makes it a viable option for resource-intensive tasks. This study not only reaffirms the importance of considering processing speed in the selection of analytical methods, but also contributes valuable insights to the field of data analysis, underscoring the importance of using statistical techniques for efficient data-driven solutions in various contexts. This study highlights the effectiveness of statistical methods in the rapid analysis of data, which is essential for decision-making and real-time applications in IoT solutions and cybersecurity. The rapid processing capabilities of the statistical classifier make it an ideal choice for tasks that require fast and efficient data processing, highlighting the critical role of processing speed in the selection of analytical methods. These results support the use of statistical techniques to improve communications security and device authentication. By demonstrating that RFFs, as defined by PDFs, can robustly identify and classify devices, this research offers a transformative strategy for strengthening the security of the IoT ecosystem. The ability to quickly and accurately identify devices through their RFFs significantly strengthens the security framework of IoT networks, reducing the risk of unauthorized access and ensuring secure data transmission. This approach not only reaffirms the importance of statistical analysis for efficient, data-driven solutions across multiple domains, but also marks a significant advancement in securing increasingly pervasive IoT infrastructures against cyber threats.

## Figures and Tables

**Table 1:** Smartphone classes and models in the case study extracted from database compiled in 2020 by Uzundurukan et al. [16].

Class Number	Smartphone Name
1	iPhone 5(1)
2	iPhone 5(2)
3	iPhone 6(1)
4	iPhone 6(2)
5	iPhone 5s(1)
6	iPhone 5s(2)
7	iPhone 6s(1)
8	iPhone 6s(1)
9	LG G4(1)
10	LG G4(2)
11	Samsung Note3(1)
12	Samsung Note3(2)
13	Samsung S5(1)
14	Samsung S5(2)
15	Sony Xperia M5(1)
16	Sony Xperia M5(2)

**Table 2:** Confusion matrix from the results obtained using the proposed method.

		Predicted Device															
		**1**	**2**	**3**	**4**	**5**	**6**	**7**	**8**	**9**	**10**	**11**	**12**	**13**	**14**	**15**	**16**
Real device	1	1.00	0.00	0.00	0.00	0.00	0.00	0.00	0.00	0.00	0.00	0.00	0.00	0.00	0.00	0.00	0.00
	2	0.00	1.00	0.00	0.00	0.00	0.00	0.00	0.00	0.00	0.00	0.00	0.00	0.00	0.00	0.00	0.00
	3	0.00	0.00	1.00	0.00	0.00	0.00	0.00	0.00	0.00	0.00	0.00	0.00	0.00	0.00	0.00	0.00
	4	0.00	0.00	0.00	1.00	0.00	0.00	0.00	0.00	0.00	0.00	0.00	0.00	0.00	0.00	0.00	0.00
	5	0.00	0.00	0.00	0.00	1.00	0.00	0.00	0.00	0.00	0.00	0.00	0.00	0.00	0.00	0.00	0.00
	6	0.00	0.00	0.00	0.00	0.00	1.00	0.00	0.00	0.00	0.00	0.00	0.00	0.00	0.00	0.00	0.00
	7	0.00	0.00	0.00	0.00	0.00	0.00	1.00	0.00	0.00	0.00	0.00	0.08	0.00	0.00	0.00	0.00
	8	0.00	0.00	0.00	0.00	0.00	0.00	0.00	1.00	0.00	0.00	0.00	0.00	0.00	0.00	0.00	0.00
	9	0.00	0.00	0.00	0.00	0.00	0.000	0.00	0.00	1.00	0.00	0.00	0.00	0.00	0.00	0.00	0.00
	10	0.00	0.00	0.00	0.00	0.00	0.00	0.00	0.00	0.00	1.00	0.00	0.00	0.00	0.00	0.00	0.00
	11	0.00	0.00	0.00	0.00	0.00	0.00	0.00	0.00	0.00	0.00	1.00	0.00	0.00	0.00	0.00	0.00
	12	0.00	0.00	0.00	0.00	0.00	0.00	0.00	0.00	0.00	0.00	0.00	0.92	0.00	0.00	0.00	0.00
	13	0.00	0.00	0.00	0.00	0.00	0.00	0.00	0.00	0.00	0.00	0.00	0.00	1.00	0.00	0.00	0.00
	14	0.00	0.00	0.00	0.00	0.00	0.00	0.00	0.00	0.00	0.00	0.00	0.00	0.00	1.00	0.00	0.00
	15	0.00	0.00	0.00	0.00	0.00	0.00	0.00	0.00	0.00	0.00	0.00	0.00	0.00	0.00	1.00	0.00
	16	0.00	0.00	0.00	0.00	0.00	0.00	0.00	0.00	0.00	0.00	0.00	0.00	0.00	0.00	0.00	1.00

**Table 3:** Confusion matrix from the results of Uzundurukan’s method.

		Predicted Device															
		**1**	**2**	**3**	**4**	**5**	**6**	**7**	**8**	**9**	**10**	**11**	**12**	**13**	**14**	**15**	**16**
Real device	1	0.57	0.00	0.00	0.00	0.00	0.03	0.00	0.00	0.00	0.00	0.00	0.17	0.00	0.00	0.03	0.00
	2	0.00	0.13	0.00	0.00	0.00	0.00	0.00	0.00	0.10	0.00	0.00	0.00	0.03	0.00	0.00	0.00
	3	0.03	0.00	0.97	0.03	0.03	0.00	0.00	0.00	0.00	0.03	0.00	0.03	0.00	0.00	0.00	0.00
	4	0.00	0.03	0.03	0.77	0.00	0.00	0.00	0.00	0.00	0.37	0.00	0.00	0.00	0.00	0.00	0.00
	5	0.00	0.00	0.00	0.00	0.90	0.10	0.03	0.00	0.00	0.00	0.00	0.00	0.00	0.00	0.00	0.00
	6	0.10	0.00	0.00	0.00	0.03	0.77	0.00	0.00	0.00	0.00	0.00	0.00	0.00	0.00	0.10	0.00
	7	0.00	0.00	0.00	0.00	0.00	0.00	0.93	0.00	0.00	0.00	0.00	0.13	0.00	0.00	0.00	0.00
	8	0.00	0.00	0.00	0.00	0.00	0.00	0.00	1.00	0.00	0.00	0.00	0.00	0.00	0.00	0.00	0.00
	9	0.00	0.77	0.00	0.03	0.03	0.00	0.00	0.00	0.87	0.10	0.00	0.00	0.10	0.00	0.00	0.00
	10	0.00	0.00	0.00	0.17	0.00	0.00	0.00	0.00	0.03	0.50	0.00	0.00	0.00	0.00	0.00	0.00
	11	0.03	0.00	0.00	0.00	0.00	0.00	0.00	0.00	0.00	0.00	1.00	0.00	0.00	0.00	0.00	0.00
	12	0.23	0.00	0.00	0.00	0.00	0.00	0.03	0.00	0.00	0.00	0.00	0.67	0.00	0.00	0.00	0.00
	13	0.00	0.07	0.00	0.00	0.00	0.03	0.00	0.00	0.00	0.00	0.00	0.00	0.87	0.00	0.00	0.00
	14	0.00	0.00	0.00	0.00	0.00	0.00	0.00	0.00	0.00	0.00	0.00	0.00	0.00	1.00	0.00	0.00
	15	0.03	0.00	0.00	0.00	0.00	0.07	0.00	0.00	0.00	0.00	0.00	0.00	0.00	0.00	0.87	0.00
	16	0.00	0.00	0.00	0.00	0.00	0.00	0.00	0.00	0.00	0.00	0.00	0.00	0.00	0.00	0.00	1.00

## Data Availability

Data will be made available on request.
